# Arrest of Development of Large Intestine and Terminal Portion of Small Intestine

**Published:** 1913-11

**Authors:** 


ARREST OF DEVELOPMENT OF LARGE INTESTINE AND TERMINAL PORTION OF SMALL INTESTINE. Marc Perrin of Paris, *La Clin.*

*Infantile,* March, 1913, mentions a case in an infant three days old on which he was called to operate for intestinal obstruction, making an artificial anus in what he supposed to be the cæcum. At necropsy it was found that the intestine, normal at the pylorus, gradually became dilated till, at 50 c.m. from the ileocæcal valve, it suddenly assumed the size of a pencil with a filiform lumen., the failure of development continuing till near the anus.

